# Bis[benzyl *N*′-(1*H*-indol-3-ylmethyl­ene)hydrazinecarbodithio­ato-κ^2^ *N*′,*S*]nickel(II) *N*,*N*-dimethyl­formamide disolvate

**DOI:** 10.1107/S1600536808038580

**Published:** 2008-11-26

**Authors:** Hamid Khaledi, Hapipah Mohd Ali, Seik Weng Ng

**Affiliations:** a Department of Chemistry, University of Malaya, 50603 Kuala Lumpur, Malaysia

## Abstract

In the title compound, [Ni(C_17_H_14_N_3_S_2_)_2_]·2C_3_H_7_NO, the Ni atom (site symmetry 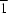) is *N*,*S*-chelated by two deprotonated Schiff base anions in a distorted square-planar geometry. The dihedral angle between the aromatic ring planes within the ligand is 86.37 (13)°. In the crystal structure, an N—H⋯O hydrogen bond links the complex to the dimethyl­formamide solvent mol­ecule.

## Related literature

For other square-planar nickel dithio­carbaza­tes, see: Ali *et al.* (2000 ▶); Tian *et al.* (1996*a* ▶,*b* ▶); Xue *et al.* (2003 ▶); Zhang *et al.* (2004 ▶); Zhu *et al.* (2000 ▶).
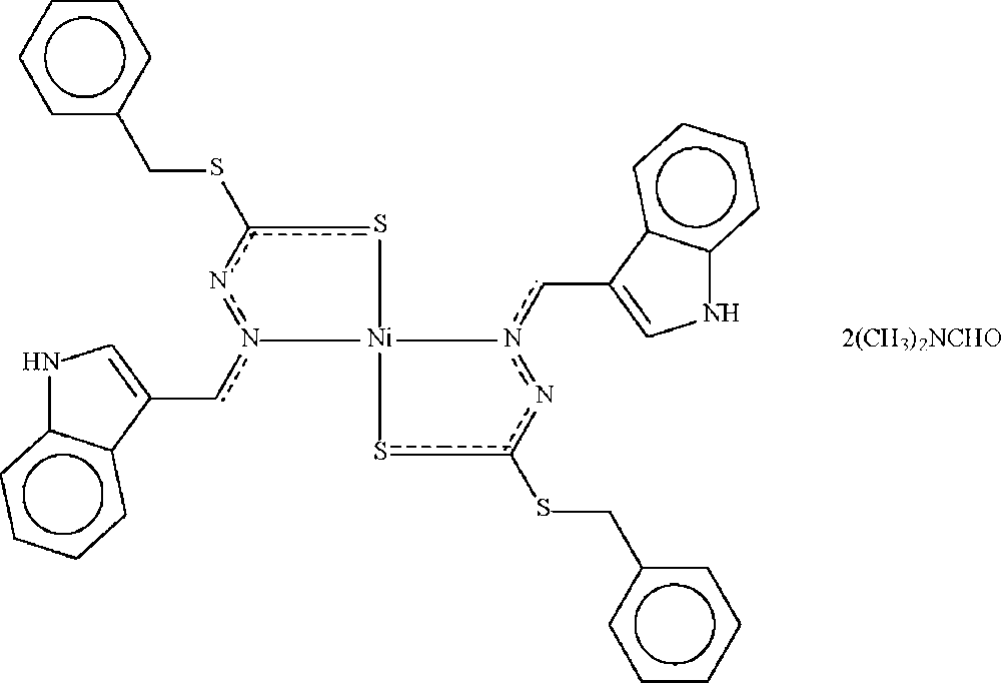


## Experimental

### 

#### Crystal data

[Ni(C_17_H_14_N_3_S_2_)_2_]·2C_3_H_7_NO
*M* *_r_* = 853.77
Monoclinic, 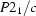

*a* = 10.3808 (3) Å
*b* = 20.0219 (7) Å
*c* = 10.7831 (3) Å
β = 117.921 (2)°
*V* = 1980.3 (1) Å^3^
*Z* = 2
Mo *K*α radiation
μ = 0.75 mm^−1^
*T* = 100 (2) K
0.12 × 0.12 × 0.06 mm

#### Data collection

Bruker SMART APEX CCD diffractometer
Absorption correction: multi-scan (*SADABS*; Sheldrick, 1996 ▶) *T*_min_ = 0.916, *T*_max_ = 0.957
13342 measured reflections
3481 independent reflections
2615 reflections with *I* > 2σ(*I*)
*R*_int_ = 0.062

#### Refinement

*R*[*F* ^2^ > 2σ(*F* ^2^)] = 0.040
*wR*(*F* ^2^) = 0.094
*S* = 1.03
3481 reflections
252 parameters
H-atom parameters constrained
Δρ_max_ = 0.50 e Å^−3^
Δρ_min_ = −0.29 e Å^−3^

### 

Data collection: *APEX2* (Bruker, 2007 ▶); cell refinement: *SAINT* (Bruker, 2007 ▶); data reduction: *SAINT*; program(s) used to solve structure: *SHELXS97* (Sheldrick, 2008 ▶); program(s) used to refine structure: *SHELXL97* (Sheldrick, 2008 ▶); molecular graphics: *X-SEED* (Barbour, 2001 ▶); software used to prepare material for publication: *pubCIF* (Westrip, 2008 ▶).

## Supplementary Material

Crystal structure: contains datablocks global, I. DOI: 10.1107/S1600536808038580/hb2849sup1.cif

Structure factors: contains datablocks I. DOI: 10.1107/S1600536808038580/hb2849Isup2.hkl

Additional supplementary materials:  crystallographic information; 3D view; checkCIF report

## Figures and Tables

**Table 1:** Selected bond lengths (Å)

Ni1—N2	1.916 (2)
Ni1—S1	2.1770 (7)

**Fig. 1.**
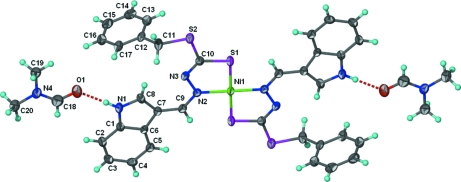
View of (I) at the 70% probability level. Hydrogen atoms are drawn as spheres of arbitrary radius. The unlabelled atoms are generated by the symmetry operation (1–x, 1–y, 1–z) and the hydrogen bonds are shown as dashed lines.

## supplementary crystallographic information

### Comment

For related structures, see: Ali *et al.* (2000); Tian *et al.* (1996*a*,*b*); Xue *et al.* (2003); Zhang *et al.* (2004); Zhu *et al.* (2000).

### Experimental

Benzyl (1*H*-indol-2-ylmethylene)hydrazinecarbodithioate ethanol hemisolvate (2 mmol, 0.65 g) was dissolved in ethanol (30 ml) along with several drops of triethylamine. To the resulting clear solution was added an ethanol solution (10 ml) containing 1 mmol (0.25 g) of nickel acetate tetrahydrate. The mixture was heated for an hour.  The product that separated was recrystallized from DMF to yield brown blocks of (I).

### Refinement

Hydrogen atoms were placed at calculated positions (C–H = 0.95–0.99Å, N–H = 0.88Å) and refined as riding with *U*(H) = 1.2–1.5 times *U*_eq_(*C*,*N*).

### Crystal data

**Table:**

[Ni(C_17_H_14_N_3_S_2_)_2_]·2C_3_H_7_NO	*F*_000_ = 892
*M**_r_* = 853.77	*D*_x_ = 1.432 Mg m^−^^3^
Monoclinic, *P*2_1_/*c*	Mo *K*α radiation λ = 0.71073 Å
Hall symbol: -P 2ybc	Cell parameters from 1450 reflections
*a* = 10.3808 (3) Å	θ = 2.2–22.7º
*b* = 20.0219 (7) Å	µ = 0.75 mm^−^^1^
*c* = 10.7831 (3) Å	*T* = 100 (2) K
β = 117.921 (2)º	Block, brown
*V* = 1980.3 (1) Å^3^	0.12 × 0.12 × 0.06 mm
*Z* = 2	

### Data collection

**Table:**

Bruker SMART APEX CCD diffractometer	3481 independent reflections
Radiation source: fine-focus sealed tube	2615 reflections with *I* > 2σ(*I*)
Monochromator: graphite	*R*_int_ = 0.062
*T* = 100(2) K	θ_max_ = 25.0º
ω scans	θ_min_ = 2.0º
Absorption correction: Multi-scan(SADABS; Sheldrick, 1996)	*h* = −12→12
*T*_min_ = 0.916, *T*_max_ = 0.957	*k* = −23→23
13342 measured reflections	*l* = −12→12

### Refinement

**Table:**

Refinement on *F*^2^	Secondary atom site location: difference Fourier map
Least-squares matrix: full	Hydrogen site location: inferred from neighbouring sites
*R*[*F*^2^ > 2σ(*F*^2^)] = 0.040	H-atom parameters constrained
*wR*(*F*^2^) = 0.094	*w* = 1/[σ^2^(*F*_o_^2^) + (0.0396*P*)^2^ + 0.2888*P*] where *P* = (*F*_o_^2^ + 2*F*_c_^2^)/3
*S* = 1.03	(Δ/σ)_max_ = 0.001
3481 reflections	Δρ_max_ = 0.50 e Å^−^^3^
252 parameters	Δρ_min_ = −0.29 e Å^−^^3^
Primary atom site location: structure-invariant direct methods	Extinction correction: none

### Fractional atomic coordinates and isotropic or equivalent isotropic displacement parameters (Å^2^)

**Table:**

	*x*	*y*	*z*	*U*_iso_*/*U*_eq_	
Ni1	0.5000	0.5000	0.5000	0.01710 (15)	
S1	0.48640 (8)	0.60841 (4)	0.48343 (8)	0.0236 (2)	
S2	0.38426 (8)	0.70431 (4)	0.62163 (8)	0.0234 (2)	
O1	0.1755 (3)	0.56685 (12)	1.1091 (2)	0.0472 (7)	
N1	0.2818 (3)	0.49406 (11)	0.9618 (2)	0.0220 (6)	
H1	0.2520	0.5191	1.0105	0.026*	
N2	0.4331 (2)	0.51020 (11)	0.6371 (2)	0.0182 (5)	
N3	0.3953 (2)	0.57357 (11)	0.6685 (2)	0.0192 (5)	
N4	0.0849 (3)	0.57529 (12)	1.2634 (2)	0.0252 (6)	
C1	0.2942 (3)	0.42563 (14)	0.9707 (3)	0.0192 (6)	
C2	0.2658 (3)	0.38131 (15)	1.0548 (3)	0.0232 (7)	
H2	0.2351	0.3967	1.1200	0.028*	
C3	0.2842 (3)	0.31463 (15)	1.0393 (3)	0.0260 (7)	
H3	0.2647	0.2832	1.0945	0.031*	
C4	0.3308 (3)	0.29162 (15)	0.9445 (3)	0.0250 (7)	
H4	0.3412	0.2450	0.9354	0.030*	
C5	0.3621 (3)	0.33626 (15)	0.8636 (3)	0.0219 (7)	
H5	0.3955	0.3205	0.8005	0.026*	
C6	0.3437 (3)	0.40438 (14)	0.8763 (3)	0.0187 (6)	
C7	0.3625 (3)	0.46401 (14)	0.8099 (3)	0.0188 (6)	
C8	0.3220 (3)	0.51697 (15)	0.8678 (3)	0.0223 (7)	
H8	0.3227	0.5627	0.8443	0.027*	
C9	0.4138 (3)	0.46239 (15)	0.7091 (3)	0.0198 (7)	
H9	0.4382	0.4191	0.6907	0.024*	
C10	0.4193 (3)	0.62076 (14)	0.6009 (3)	0.0191 (6)	
C11	0.3353 (3)	0.70343 (15)	0.7620 (3)	0.0245 (7)	
H11A	0.4028	0.6726	0.8354	0.029*	
H11B	0.3535	0.7487	0.8039	0.029*	
C12	0.1817 (3)	0.68378 (14)	0.7277 (3)	0.0206 (7)	
C13	0.0673 (3)	0.68232 (15)	0.5927 (3)	0.0259 (7)	
H13	0.0838	0.6939	0.5159	0.031*	
C14	−0.0716 (3)	0.66398 (16)	0.5687 (3)	0.0309 (8)	
H14	−0.1490	0.6622	0.4754	0.037*	
C15	−0.0977 (3)	0.64846 (16)	0.6793 (3)	0.0320 (8)	
H15	−0.1930	0.6364	0.6627	0.038*	
C16	0.0152 (3)	0.65049 (15)	0.8139 (3)	0.0291 (8)	
H16	−0.0020	0.6398	0.8908	0.035*	
C17	0.1540 (3)	0.66816 (15)	0.8379 (3)	0.0243 (7)	
H17	0.2312	0.6696	0.9313	0.029*	
C18	0.1381 (4)	0.54196 (18)	1.1919 (3)	0.0376 (9)	
H18	0.1485	0.4950	1.2054	0.045*	
C19	0.0701 (3)	0.64730 (15)	1.2501 (3)	0.0289 (8)	
H19A	0.0755	0.6616	1.1658	0.043*	
H19B	0.1489	0.6684	1.3329	0.043*	
H19C	−0.0241	0.6606	1.2427	0.043*	
C20	0.0490 (3)	0.54232 (15)	1.3634 (3)	0.0293 (7)	
H20A	0.0590	0.4939	1.3579	0.044*	
H20B	−0.0516	0.5531	1.3412	0.044*	
H20C	0.1154	0.5578	1.4584	0.044*	

### Atomic displacement parameters (Å^2^)

**Table:**

	*U*^11^	*U*^22^	*U*^33^	*U*^12^	*U*^13^	*U*^23^
Ni1	0.0178 (3)	0.0178 (3)	0.0192 (3)	−0.0001 (2)	0.0116 (2)	−0.0001 (2)
S1	0.0326 (5)	0.0197 (4)	0.0292 (4)	0.0005 (3)	0.0234 (4)	0.0002 (3)
S2	0.0297 (5)	0.0183 (4)	0.0296 (4)	−0.0010 (3)	0.0201 (4)	−0.0016 (3)
O1	0.0752 (19)	0.0443 (17)	0.0405 (14)	0.0204 (13)	0.0424 (14)	0.0058 (12)
N1	0.0274 (14)	0.0212 (15)	0.0242 (13)	−0.0007 (11)	0.0178 (11)	−0.0028 (11)
N2	0.0186 (13)	0.0152 (14)	0.0220 (12)	0.0001 (10)	0.0106 (10)	−0.0005 (10)
N3	0.0197 (13)	0.0175 (14)	0.0223 (13)	0.0009 (10)	0.0114 (11)	−0.0031 (10)
N4	0.0265 (15)	0.0263 (16)	0.0239 (13)	0.0015 (11)	0.0129 (12)	−0.0056 (11)
C1	0.0192 (16)	0.0213 (17)	0.0180 (14)	−0.0012 (13)	0.0094 (12)	0.0003 (12)
C2	0.0225 (17)	0.0289 (19)	0.0212 (15)	−0.0016 (14)	0.0128 (13)	0.0023 (13)
C3	0.0232 (17)	0.030 (2)	0.0264 (16)	0.0009 (14)	0.0128 (14)	0.0100 (14)
C4	0.0229 (17)	0.0197 (17)	0.0319 (17)	0.0021 (13)	0.0123 (14)	0.0079 (13)
C5	0.0188 (16)	0.0253 (18)	0.0229 (15)	0.0023 (13)	0.0110 (13)	0.0003 (13)
C6	0.0129 (15)	0.0237 (18)	0.0205 (15)	0.0019 (12)	0.0088 (12)	0.0023 (12)
C7	0.0172 (16)	0.0200 (17)	0.0203 (15)	−0.0010 (12)	0.0098 (13)	−0.0001 (12)
C8	0.0243 (17)	0.0242 (18)	0.0217 (15)	−0.0019 (13)	0.0134 (13)	0.0033 (12)
C9	0.0172 (16)	0.0201 (18)	0.0237 (16)	0.0008 (12)	0.0107 (13)	−0.0002 (12)
C10	0.0162 (16)	0.0222 (17)	0.0183 (14)	−0.0012 (12)	0.0076 (12)	−0.0008 (12)
C11	0.0304 (18)	0.0232 (18)	0.0263 (16)	−0.0054 (14)	0.0186 (14)	−0.0074 (13)
C12	0.0251 (17)	0.0148 (16)	0.0282 (16)	0.0002 (13)	0.0177 (14)	−0.0055 (12)
C13	0.0301 (19)	0.0247 (18)	0.0265 (17)	0.0025 (14)	0.0164 (15)	−0.0007 (13)
C14	0.0224 (18)	0.032 (2)	0.0314 (18)	0.0053 (14)	0.0071 (15)	−0.0008 (15)
C15	0.0232 (18)	0.031 (2)	0.044 (2)	0.0045 (14)	0.0177 (16)	0.0048 (15)
C16	0.0322 (19)	0.0273 (19)	0.0366 (19)	0.0018 (14)	0.0234 (16)	0.0042 (14)
C17	0.0246 (18)	0.0256 (18)	0.0264 (16)	−0.0007 (14)	0.0150 (14)	−0.0043 (13)
C18	0.048 (2)	0.034 (2)	0.0333 (19)	0.0081 (17)	0.0206 (18)	−0.0021 (16)
C19	0.034 (2)	0.028 (2)	0.0260 (17)	0.0037 (15)	0.0150 (15)	−0.0037 (14)
C20	0.0300 (18)	0.029 (2)	0.0324 (18)	−0.0026 (14)	0.0177 (15)	−0.0049 (14)

### Geometric parameters (Å, °)

**Table:**

Ni1—N2^i^	1.916 (2)	C6—C7	1.451 (4)
Ni1—N2	1.916 (2)	C7—C8	1.391 (4)
Ni1—S1	2.1770 (7)	C7—C9	1.418 (4)
Ni1—S1^i^	2.1770 (7)	C8—H8	0.9500
S1—C10	1.725 (3)	C9—H9	0.9500
S2—C10	1.748 (3)	C11—C12	1.511 (4)
S2—C11	1.808 (3)	C11—H11A	0.9900
O1—C18	1.233 (4)	C11—H11B	0.9900
N1—C8	1.346 (3)	C12—C17	1.383 (4)
N1—C1	1.375 (3)	C12—C13	1.382 (4)
N1—H1	0.8800	C13—C14	1.390 (4)
N2—C9	1.306 (3)	C13—H13	0.9500
N2—N3	1.416 (3)	C14—C15	1.377 (4)
N3—C10	1.287 (3)	C14—H14	0.9500
N4—C18	1.321 (4)	C15—C16	1.375 (4)
N4—C20	1.454 (4)	C15—H15	0.9500
N4—C19	1.450 (4)	C16—C17	1.385 (4)
C1—C2	1.395 (4)	C16—H16	0.9500
C1—C6	1.404 (4)	C17—H17	0.9500
C2—C3	1.370 (4)	C18—H18	0.9500
C2—H2	0.9500	C19—H19A	0.9800
C3—C4	1.398 (4)	C19—H19B	0.9800
C3—H3	0.9500	C19—H19C	0.9800
C4—C5	1.390 (4)	C20—H20A	0.9800
C4—H4	0.9500	C20—H20B	0.9800
C5—C6	1.393 (4)	C20—H20C	0.9800
C5—H5	0.9500		
			
N2^i^—Ni1—N2	180.0	C7—C9—H9	114.5
N2^i^—Ni1—S1	94.36 (7)	N3—C10—S1	124.2 (2)
N2—Ni1—S1	85.64 (7)	N3—C10—S2	121.5 (2)
N2^i^—Ni1—S1^i^	85.64 (7)	S1—C10—S2	114.26 (16)
N2—Ni1—S1^i^	94.36 (7)	C12—C11—S2	118.4 (2)
S1—Ni1—S1^i^	180.0	C12—C11—H11A	107.7
C10—S1—Ni1	96.64 (10)	S2—C11—H11A	107.7
C10—S2—C11	104.76 (13)	C12—C11—H11B	107.7
C8—N1—C1	109.9 (2)	S2—C11—H11B	107.7
C8—N1—H1	125.1	H11A—C11—H11B	107.1
C1—N1—H1	125.1	C17—C12—C13	118.5 (3)
C9—N2—N3	112.2 (2)	C17—C12—C11	118.0 (2)
C9—N2—Ni1	126.4 (2)	C13—C12—C11	123.5 (3)
N3—N2—Ni1	121.46 (17)	C12—C13—C14	120.4 (3)
C10—N3—N2	111.9 (2)	C12—C13—H13	119.8
C18—N4—C20	121.8 (3)	C14—C13—H13	119.8
C18—N4—C19	119.9 (3)	C15—C14—C13	120.4 (3)
C20—N4—C19	118.2 (2)	C15—C14—H14	119.8
N1—C1—C2	129.6 (3)	C13—C14—H14	119.8
N1—C1—C6	107.8 (2)	C14—C15—C16	119.4 (3)
C2—C1—C6	122.6 (3)	C14—C15—H15	120.3
C3—C2—C1	117.2 (3)	C16—C15—H15	120.3
C3—C2—H2	121.4	C15—C16—C17	120.2 (3)
C1—C2—H2	121.4	C15—C16—H16	119.9
C2—C3—C4	121.7 (3)	C17—C16—H16	119.9
C2—C3—H3	119.2	C12—C17—C16	121.0 (3)
C4—C3—H3	119.2	C12—C17—H17	119.5
C5—C4—C3	120.7 (3)	C16—C17—H17	119.5
C5—C4—H4	119.7	O1—C18—N4	125.3 (3)
C3—C4—H4	119.7	O1—C18—H18	117.3
C4—C5—C6	119.0 (3)	N4—C18—H18	117.3
C4—C5—H5	120.5	N4—C19—H19A	109.5
C6—C5—H5	120.5	N4—C19—H19B	109.5
C5—C6—C1	118.8 (3)	H19A—C19—H19B	109.5
C5—C6—C7	134.5 (3)	N4—C19—H19C	109.5
C1—C6—C7	106.7 (2)	H19A—C19—H19C	109.5
C8—C7—C9	131.5 (3)	H19B—C19—H19C	109.5
C8—C7—C6	105.5 (2)	N4—C20—H20A	109.5
C9—C7—C6	123.0 (3)	N4—C20—H20B	109.5
N1—C8—C7	110.1 (3)	H20A—C20—H20B	109.5
N1—C8—H8	125.0	N4—C20—H20C	109.5
C7—C8—H8	125.0	H20A—C20—H20C	109.5
N2—C9—C7	131.1 (3)	H20B—C20—H20C	109.5
N2—C9—H9	114.5		
			
N2^i^—Ni1—S1—C10	177.84 (11)	C9—C7—C8—N1	−179.2 (3)
N2—Ni1—S1—C10	−2.16 (11)	C6—C7—C8—N1	0.2 (3)
S1—Ni1—N2—C9	−177.9 (2)	N3—N2—C9—C7	0.1 (4)
S1^i^—Ni1—N2—C9	2.1 (2)	Ni1—N2—C9—C7	−178.5 (2)
S1—Ni1—N2—N3	3.52 (18)	C8—C7—C9—N2	−2.8 (5)
S1^i^—Ni1—N2—N3	−176.48 (18)	C6—C7—C9—N2	177.9 (3)
C9—N2—N3—C10	177.8 (2)	N2—N3—C10—S1	1.1 (3)
Ni1—N2—N3—C10	−3.5 (3)	N2—N3—C10—S2	−179.25 (17)
C8—N1—C1—C2	179.3 (3)	Ni1—S1—C10—N3	1.2 (2)
C8—N1—C1—C6	−0.5 (3)	Ni1—S1—C10—S2	−178.47 (13)
N1—C1—C2—C3	178.4 (3)	C11—S2—C10—N3	6.4 (3)
C6—C1—C2—C3	−1.9 (4)	C11—S2—C10—S1	−173.95 (15)
C1—C2—C3—C4	0.7 (4)	C10—S2—C11—C12	−80.3 (2)
C2—C3—C4—C5	0.8 (4)	S2—C11—C12—C17	165.3 (2)
C3—C4—C5—C6	−1.2 (4)	S2—C11—C12—C13	−16.5 (4)
C4—C5—C6—C1	0.0 (4)	C17—C12—C13—C14	−1.5 (4)
C4—C5—C6—C7	−178.9 (3)	C11—C12—C13—C14	−179.7 (3)
N1—C1—C6—C5	−178.6 (2)	C12—C13—C14—C15	1.4 (5)
C2—C1—C6—C5	1.5 (4)	C13—C14—C15—C16	−0.7 (5)
N1—C1—C6—C7	0.6 (3)	C14—C15—C16—C17	0.1 (5)
C2—C1—C6—C7	−179.3 (3)	C13—C12—C17—C16	0.9 (4)
C5—C6—C7—C8	178.6 (3)	C11—C12—C17—C16	179.2 (3)
C1—C6—C7—C8	−0.5 (3)	C15—C16—C17—C12	−0.2 (5)
C5—C6—C7—C9	−2.0 (5)	C20—N4—C18—O1	−177.4 (3)
C1—C6—C7—C9	179.0 (2)	C19—N4—C18—O1	−1.6 (5)
C1—N1—C8—C7	0.2 (3)		

Symmetry codes: (i) −*x*+1, −*y*+1, −*z*+1.

### Hydrogen-bond geometry (Å, °)

**Table:**

*D*—H···*A*	*D*—H	H···*A*	*D*···*A*	*D*—H···*A*
N1—H1···O1	0.88	1.86	2.739 (3)	175

## References

[bb1] Ali, M. A., Mirza, A., Butcher, R. J. & Rahman, M. (2000). *Transition Met. Chem.* **25**, 430–436.

[bb11] Barbour, L. J. (2001). *J. Supramol. Chem.* **1**, 189–191.

[bb2] Bruker (2007). *APEX2* and *SAINT* Bruker AXS Inc., Madison, Wisconsin, USA.

[bb3] Sheldrick, G. M. (1996). *SADABS* University of Göttingen, Germany.

[bb4] Sheldrick, G. M. (2008). *Acta Cryst.* A**64**, 112–122. 10.1107/S0108767307043930 18156677

[bb5] Tian, Y.-P., Duan, C.-Y., Lu, Z.-L., You, X.-Z., Fun, H.-K. & Kandasamy, S. (1996*a*). *Polyhedron*, **15**, 2263–2271.

[bb6] Tian, Y.-P., Duan, C.-Y., Lu, Z.-L., You, X.-Z. & Huang, X.-Y. (1996*b*). *J. Coord. Chem.* **38**, 219–226.

[bb7] Westrip, S. P. (2008). *publCIF* In preparation.

[bb8] Xue, Z.-M., Zhang, X.-J., Tian, Y.-P., Wu, J.-Y., Jiang, M.-H. & Fun, H.-K. (2003). *Chin. J. Struct. Chem.* **22**, 265–269.

[bb9] Zhang, M.-L., Tian, Y.-P., Zhang, X.-J., Wu, J.-Y., Zhang, S.-Y., Wang, D., Jiang, M.-H., Chantrapromma, S. & Fun, H.-K. (2004). *Transition Met. Chem.* **29**, 596–602.

[bb10] Zhu, X.-H., Chen, X.-F., Zhang, Y., You, X.-Z., Tan, W.-L., Ji, W., Vittal, J. J., Tan, G.-K. & Kennard, C. H. L. (2000). *New J. Chem.* **24**, 419–423.

